# Corruption red flags in public procurement: new evidence from Italian calls for tenders

**DOI:** 10.1140/epjds/s13688-022-00325-x

**Published:** 2022-03-18

**Authors:** Francesco Decarolis, Cristina Giorgiantonio

**Affiliations:** 1 Department of Economics, Bocconi University, Milano, Italy; 2 IGIER, Milano, Italy; 3 Research Department, Bank of Italy, Rome, Italy

**Keywords:** D44, D47, H57, R42, Public procurement, Corruption, Red flags

## Abstract

This paper contributes to the analysis of quantitative indicators (i.e., *red flags* or *screens*) to detect corruption in public procurement. It presents an approach to evaluate corruption risk in public tenders through standardized ML tools applied to detailed data on the content of calls for tenders. The method is applied to roadwork contracts in Italy and three main contributions are reported. First, the study expands the set of commonly discussed indicators in the literature to new ones derived from operative practices of police forces and the judiciary. Second, using novel and unique data on firm-level corruption risk, this study validates the effectiveness of the indicators. Third, it quantifies the increased corruption-prediction ability when indicators that are known to be unavailable to the corruption-monitoring authority are included in the prediction exercise. Regarding the specific red flags, we find a systematic association between high corruption risk and the use of multi-parameter awarding criteria. Furthermore, predictability of the red flag makes them ineffective as prediction tools: the most obvious and scrutinized red flags are either uncorrelated with corruption or, even, negatively associated with it, as it is the case for invoking special procedures due to “urgency,” or the extent of publicity of the call for tender.

## Introduction

Corruption is commonly defined as the abuse of public power to obtain private benefits. It is widely believed to entail high economic and social costs. Its importance for economic growth has been of policy interest to governments, entrepreneurs, and investors around the world, with the IMF estimating that corruption costs exceed 2% of the world’s GDP (IMF [39]).

The economic literature has so far explored several channels through which corruption may affect economic growth and allocative efficiency. Some authors argue that corruption acts as a sand in the wheel and hampers economic growth, through channels such as barriers to entrepreneurship and firm investment, limited access to finance, and higher transaction costs (Shleifer and Vishny [61], Mauro [47], Svensson [63]), resulting in resource misallocation across firms (Hsieh and Klenow [37]) and within firms (Murphy, Shleifer and Vishny [53], Dal Bó and Rossi [21], Colonnelli and Prem [19]). Others highlight its effects in terms of distortion of human capital accumulation (Mo [50]). Furthermore, some studies focus on the activities of the public sector, documenting relationships between corruption and inefficiency in the composition of government expenditure (Mauro [48]), lower productivity of public investments (Del Monte and Papagni [25]), higher shares of goods and services procured by the public administration on noncompetitive markets (Hessami [36]), worse selection and misallocation of public employees (Mocetti and Orlando [51]).^1^

Public procurement is a particularly critical area for corruption (Golden and Picci [33], ANAC [2]). Nearly all activities that involve the public sector imply the need to procure goods, services or works, from construction to education, from healthcare to innovation. However, the disconnection between who secures these contracts and who pays for them creates scope for corruption. The vulnerability of public procurement—representing a crucial area in the economy, 15% of the EU-wide GDP—to corruption is a key motive behind continuous efforts to monitor, measure, and fight such crime. Moreover, in public as well as in private procurement, corruption might be the necessary evil that comes together with empowering agents to use their discretion: curbing corruption through rigid procurement rules might impose significant efficiency losses, even higher than those resulting from corruption itself (Manelli and Vincent [46], Calzolari and Spagnolo [14]) and Decarolis et al. [22]).

In this study, we analyze how different public procurement features are associated with the risk of corruption. That is, we take an ex ante perspective and ask which features of the tendering design are best capable of predicting the risk of follow on corruption at the contract awarding stage. In doing so, we contribute to the ongoing competition economics literature of methods to detect illegal practices in the context of public procurement. While the literature is abundant in methods to test for bid rigging and collusion (see for example Porter and Zona [55] and more recently Chassang et al. [16]), corruption detection methods are much less developed. We do not seek to evaluate the trade-offs created by, for instance, bolstering flexibility in choosing the most reliable contractors relative to the risk of abusing discretion, which is the focus of the related study by Decarolis et al. [22]. Using novel data concerning the procurement of public works,^2^ in this paper, we contribute to this debate by providing new evidence on so-called “red flags”, i.e. indicators of potential corruption risk.^3^ These are identifiable features of the calls for tenders that are plausibly associated with corrupt practices. The first part of this study presents these indicators, some of which are new to the literature. Some of them derive from operating practices (e.g., investigations by the sector Authority (Italian Anticorruption Authority—ANAC) or judgments). We organize their discussion based on the type of activity that they are involved in and whether they are directly available to the Anticorruption Authority or not. This entity monitors corruption risk but does not systematically collect information on all indicators. Thus, we define *oblivious indicators* those statistics (variables) that the Authority does not specifically track, even though it collects the data needed to compute them.

Next, we introduce our outcome measures of corruption risk. A reliable measure of corruption is hard to get but crucial for indicators’ validation. We employ a novel measure based on police investigations, which is first developed by Decarolis et al. [22]. As explained in greater detail in their study, such a measure exploits uniquely detailed data on firm-level corruption risk. Indeed, it is an indicator variable measuring for each firm winning a contract whether any of its owners or top managers have been the object of a police investigation for corruption-related crimes. We show quantitatively the usefulness of this new measure by comparing it to four alternative corruption proxies that are already known to the literature. Two of them derive from judiciary cases, and the other two are inferred from economic outcomes.

We then assess the prediction capability of the various indicators using standard machine learning (ML) algorithms: LASSO, Ridge regression, and random forest (as well as OLS for comparison purposes). We analyze their performance in two datasets. The first one contains more observations but only a small set of indicators. It comes from the Anticorruption Authority and includes the all of the contracts that this Authority is in charge of monitoring (13 thousand contracts in our sample period). We observe 12 main red flags in such data. The second dataset is a partially overlapping sample of the first one but includes many more variables. It has fewer observations, as it contains nearly 3.5 thousand contract awards, but incorporates the call for tenders documents for each of these contracts. Thanks to the combination of both human and machine textual analysis of these calls for tenders, we obtain a broader set of indicators. In addition to the previous 12 indicators, we can retrieve 20 new red flags, which include the oblivious indicators, that are known by participants not to be systematically monitored.

Our main findings are as follows. First, when using the smaller set of red flags available to the Anticorruption Authority, we find a systematic association between high corruption risk and some policy-relevant tools, like employing an awarding criterion based on multiple parameters (i.e., scoring auction or most economically advantageous tender). Such an awarding criterion, starting with the EU Procurement Directive 24/2014, has become the default system in the EU, replacing the previous system which had price competition as the default mechanism.^4^ This finding is consistent with recent, cross-country evidence on corruption risk from Fazekas and Kocsis [30] where 2.8 million contracts from twenty-eight European countries are analyzed. Like them, we find that the details of the scoring criteria and also the exact text of the eligibility criteria (technical, financial and other conditions for participating) is where deliberate attempts to restrict competition are hidden.

Second, we show that some indicators, which common wisdom usually considers to be positively associated with corruption, are instead negatively correlated with it. For instance, this is the case of a call for tender invoking special procedures due to “urgency”, or the number of days firms can submit their bid following the call for tenders. We argue that it is precisely for its known corruption-related risks that corrupted agents, aware of being monitored, do not employ such obvious and scrutinized tools. For this reason, we explore the predictive contribution of the oblivious and not scrutinized indicators, which leads to our third result: once we include the broader set of indicators, the model’s accuracy (measured by the mean squared error criterion) improves. However, this improvement is limited to the random forest model, and not to the LASSO or Ridge models. In line with the literature, we argue that this is likely due to the greater functional form flexibility of the random forest relative to the other two alternative methods considered.^5^ Thus, a broader set of indicators is crucial as not only some of them may be individually relevant red flags, but they also allow ML’s prediction approaches to exploit functional form flexibility.

There are several policy implications stemming from our analysis. The first one is that improving the data collection process concerning public contract call for tenders can be a useful strategy to limit corruption. In Italy, systematic data collection of public contracts dates back to year 2000 and its scope was expanded in 2008, nevertheless many of the indicators that we found to be relevant are not currently collected. Second, some of the indicators presented in this study come from evaluation of court cases. This calls for the importance of structuring a communication flow between courts and the authority supervising the data collection process so that the list of red flags is updated and timely. Third, the evidence on the adaptation ability revealed by the opposite effects of those indicators known to be certainly monitored relative to those known to be unmonitored suggests the benefits of limiting access to the information about which features are subject to monitoring (and how the data are used in the monitoring efforts).^6^ Lastly, to summarize the evidence on the substantive findings about the specific indicators, it seems that private firm competition is a key feature to curb corruption risk. Indicators on both the ease of accessing tendering information and placing bids are systematically associated with corruption. Thus, enhancing private firm competition appears to be a powerful tool to curb corruption risks. In the conclusion, we offer more policy implications based on the evidence for the specific indicators that will be discussed below.

The remainder of the paper is organized as follows: Sect. 2 reviews the literature; Sect. 3 describes the institutional framework and presents our red flags; Sect. 4 illustrates the samples of public work contracts and provides descriptive evidence on the indicators; Sect. 5 discusses the outcome measures and the strategy for their empirical analysis; Sect. 6 presents the findings; Sect. 7 concludes.

## Literature

Understanding the effects of corruption on the (mis)allocation of resources is at the heart of the economic and political debate. However, answering the question of how we can measure the extent of corruption presents a significant challenge.

A first strand of the corruption literature to which this study contributes is the vast debate over corruption measurement, a well known complex and elusive task.^7^ We use an alternative measure for distinguishing firms at risk of corruption, first presented in a new study by Decarolis et al. [22], and compare it with alternative measures that we collect on both judicial and economic outcomes. While our main corruption measure comes from Decarolis et al. [22], our contribution is clearly distinct from theirs along three dimensions. First, our research question regards red flags, while they look at evaluating the trade-offs of discretion in procurement. Second, our approach is based on predictive ML methods, while their involves ex post, causal evaluation. Third, our data collected from calls for tenders is original to this study.

Unlike the subjective measures often used in the corruption literature such as corruption indexes,^8^ our measure has the merit of being an objective way to measure corruption. However, it is superior to other objective measures such as the ones based on judicial data, as these only measure emerged corruption (e.g., convictions include only acts of corruption of caught and convicted individuals). Moreover, the extent to which the judiciary successfully prosecutes corruption crimes depends on several factors, including the enforcement level. Both these considerations explain why indicators based on judicial data are rarely considered an accurate measure of corruption.^9^ In light of the limitations of the judiciary data, the most recent economic literature has moved towards developing new and more objective tools to assess the extent of corruption. Some studies have employed direct measurements of outcomes (Di Tella and Schargrodsky [27], Bandiera, Prat and Valletti [5], Golden and Picci [33]). However, we show that the most often utilized indirect measures of corruption (delays and cost overruns) are not useful proxies for corruption in our context.^10^

Another part of the corruption literature to which our study is closely connected is the one on red flags in public procurement. Researchers have discussed indicators or red flags that point to evidence of corruption, which could constitute the basis for an indicator-based risk assessment (Di Nicola and McCallister [26]). The potential usefulness of red flags for detecting corruption entails specific forms of economic behavior (e.g., low bid participation rates, inexplicably wealthy public officials, poorly negotiated public procurement contracts) and that this behavior leaves traces (Kenny and Musatova [41]). Consequently, red flags are accumulations of traces that may point to the presence of corrupt activities. They may be a valuable aid for practitioners, investigators, and policymakers to estimate the corruption probability in a procurement case and to lay the foundation of a new evidence-based approach to fighting corruption.^11^ Our paper contributes to this strand of the literature in multiple ways: by proposing new indicators, validating them, and quantifying the marginal contribution of oblivious indicators.

Finally, from a methodological perspective, our use of ML algorithms is in the spirit of Kleinberg et al.’s [42] “prediction policy problems.” These are policy problems involving a prediction component, and, for them, ML techniques are likely to dominate other statistical methodologies. The use of ML algorithms can prove to be particularly useful also when researchers need to model complex relationships without having “a priori” knowledge on the exact structure of the problem. Furthermore, in the presence of data availability constraints, gains in predictive accuracy due to functional flexibility might outweigh those coming from additional data. In particular, we use off-the-shelf methods and find great improvements when using random forests to flexibly select functional forms. When assessing red flags, ML methods are useful not only because they deal with the tradeoff between the expressiveness of the model (e.g., more covariates included in linear regression) and risk of over-fitting (e.g., too many covariates relative to the sample size).^12^ Also, few red flags have a ground truth causal effect on corruption. Most red flags look at mere tools for corrupt arrangements. As these tools can be easily substituted with others, the usefulness of the red flags is closely connected with how easily such modification in corruption practices can occur. Thus, the prediction exercise is both appropriate to study red flags and a first step in the search for indicators having causal effects on corruption.

## Institutional framework and corruption indicators

In the period of our analysis (2009–2015), the regulations in place for the procurement of public works entail a highly decentralized system.^13^ Local authorities (municipalities, counties, and regions) hold the vast majority of public tenders and spend about half of the total resources allocated every year to public infrastructures, about €25 billion. In this highly fragmented system, there were about twelve thousand different purchasing authorities (PAs) active as of 2018. These PAs are heterogeneous in their tasks, capacities, and risks of being involved in corruption episodes.

Despite such heterogeneity, there is mostly a uniform set of rules that these PAs must follow to award public contracts, based on the provisions of European Directives on public procurement. As regards contracts involving higher amounts (€5million or more), PAs must procure them using competitive procedures (open or restricted participation), where all qualified firms participate. The winner is then selected either solely based on the price offered or using a scoring formula that combines points earned for the price and the technical components of the bid.^14^ This latter criterion to select the winner is known as the most economically advantageous tender (MEAT). Below the €5million threshold, PAs have more discretion in picking not only between the price-only or MEAT criterion but also alternative procedures to the open one. In particular, the smaller is the economic value of the contract up for tender, the more the PAs can restrict competition running either competitive procedures only to selected bidders or conducting a direct negotiation with one or a few bidders.^15^

In addition to the awarding criteria and procedures, PAs have discretion over other features of the call for tenders that are likely to affect the corruption risk. Beyond some minimal requirements prescribed by the law, PAs can influence two main aspects. The first is the transparency of the process. They decide both how widely to advertise the call for tenders (for instance, by advertising it online and over traditional media) and how detailed are the job descriptions disclosed to potential bidders. The second is the degree of the obstacles to participation that they can erect. While the national (and European) regulations try to curtail this margin of discretion, under certain conditions, the PAs can restrict participation to lists of trusted bidders or impose more subtle, but effective, barriers. For instance, they specify ad hoc rules for subcontracting, restricting the amount of work to the subcontractor may carry out or its identity (for instance, excluding those firms bidding in the call for tenders). Furthermore, PAs can require bidders to inspect the detailed project specs or the worksite (or both) and, simultaneously, restrict how and when these inspections can take place: nothing in the law prevents a PA from making the compulsory worksite inspection available for just a tiny window of time. These margins of discretion can serve an important role to help the PAs to achieve publicly desirable goals, but can also trigger corruption phenomena.

In the light of these considerations, we incorporate these and other elements of the call for tenders into a broad set of corruption indicators. In Table 1, we present our list of indicators along with three different dimensions that we use to classify them. The first column reports the 18 indicators, some of which have sub-indicators.^16^ The following three columns subdivide the indicators along three dimensions: type of activity that they pertain to, their accessibility to the Supervising Authority (Anticorruption Authority, ANAC), and their source being the literature or operating practices.^17^

**Table 1:** Corruption indicators: the eighteen red flags

Indicator	Sub-indicator	Activity	Accessibility	Source
*1. Absence of tender call*		Information completeness	No	OP
*2. call for tenders: page and word number*		″	No	OP
*3. ANAC info available*		″	No	OP
*4. Negotiated procedures*	*4.1 Negotiated procedure*	Awarding procedures	Yes	L/OP
	*4.2 Urgency*	″	Yes	L/OP
	*4.3 No tender*	″	Yes	L/OP
	*4.4 No t/n*	″	Yes	L/OP
*5. Legality protocols*		″	Yes	OP
*6. Local regulations*		″	No	OP
*7. Design-Build*		″	Yes	OP
*8. Scoring rule (MEAT)*	*8.1 MEAT*	Awarding criteria	Yes	L/OP
	*8.2 MEAT—Tech Score*	″	Yes	L/OP
	*8.3 MEAT—Qual. Score*	″	No	L/OP
*9. Price Only—w. ABA*		″	No	L/OP
*10. No possibility of single source award*		″	Yes	L
*11. Preferred firm indications*	*11.1 Firm list preference*	Obstacles to participation	No	L/OP
	*11.2 Firm other preference*	″	No	L/OP
*12. Open tender days (ODT)*	*12.1 OTD*	″	Yes	OP
	*12.2 OTD violation*	″	Yes	OP
*13. Document verification (DV)*	*13.1 DV*	″	No	OP
	*13.2 DV—Specific dates*	″	No	OP
	*13.3 DV—Hours share*	″	No	OP
	*13.4 DV—Hours total*	″	No	OP
*14. Worksite verification (WV)*	*14.1 WV*	″	No	OP
	*14.2 WV—Specific dates*	″	No	OP
	*14.3 WV—Hours share*	″	No	OP
	*14.4 WV—Hours total*	″	No	OP
*15. Ad hoc rules for subcontracting*	*15.1 Ad hoc rules*	″	No	L/OP
	*15.2 No subcontracting*	″	Yes	L/OP
*16. Prohibition of pooling agreements*		″	No	OP
*17. Multiple contact points*		″	No	OP
*18. External contact points*		″	No	OP

Note: refer to Appendix A for an enhanced description of each indicator.

*i*) *Source*. The first contribution of this study is indeed opening up the academic debate on some indicators previously employed only in the operating practice of the fight against corruption. We have noted earlier that the economic literature suggested an already broad set of indicators that have been either used in practice or just derived as implications of models of corruption in public contracting. We thus contribute by adding a few additional indicators that we define as originating from operating practices (OP) in Table 1. Our extensive review of the judicial authorities’ sentences on corruption in public auctions (discussed in the next section) allowed us to identify specific indicators that capture the actions of agents involved in known corruption cases but are not discussed in the literature. For example, in a large corruption scandal in the area of Naples, the judge identified that a distorted use of a provision in the call for tenders was vital for the corruption scheme:^18^ the visit to the worksite entailed the interaction with a specific individual in the PA. However, this individual was using his knowledge about firms interested in the job to inform of their identity the Camorra local clan (the Casalesi), who could then dissuade these firms from bidding. Hence, the provision of a compulsory worksite visit and the details of its working allowed the Casalesi to have full control of the public works administered by the corrupt public agent. In our analysis of calls for tender, we collect a few indicators about the worksite visit. The same we do for several other indicators in the group denoted by OP in Table 1.

*ii*) *Type of activity*. A second way in which we classify our indicators is by the type of activity they pertain to. We can distinguish four groups in which the red flags are organized: *a*) information completeness; *b*) awarding procedures; *c*) awarding criteria; *d*) obstacles to participation. To the first group belong indicators involving the transparency and publicity of the call, like the availability and completeness of the call for tenders. To the second belong those indicators specifying how the awarding procedure differs from the default open auction system, like a negotiated procedure (with or without a public call for tenders). To the third belong indicators for the awarding criterion used, which at the most aggregate level can be either a price-only criterion or a scoring rule one, weighing together price and other quantitative or qualitative technical features. The fourth group contains a large set of obstacles to participation that PAs can erect by directly limiting firms’ participation or, more indirectly, make harder through various requirements on behaviors to take ex ante (like visiting the worksite) or ex post (like limiting subcontracting). The Appendix contains a detailed discussion of each indicator.

*iii*) *Accessibility*. The last type of classification is by the indicators’ accessibility. Here we take the point of view of how readily available is the measurement of the indicator for the Anticorruption Authority. Systematic surveillance over a specific indicator requires that this indicator is among the fields that PAs have to fill in the online forms that feed the database maintained by the Anticorruption Authority.^19^ If the indicator is communicated, we consider it as accessible, otherwise not. While not discussed in the literature, we believe partitioning the indicators in such way particularly interesting. It allows us to discuss the well-known phenomenon of the elusion of monitoring efforts: when agents are aware of being monitored, they might intentionally behave not to raise suspicion. In Sect. 7, we return to this distinction to contrast the effectiveness of corruption detection with accessible and oblivious indicators.^20^

Interestingly, many of these indicators are common across the public procurement sectors of various countries. Firstly, our accessible indicators derive from fundamental elements present in all public procurement legal framework (e.g., the distinction between price-only and MEAT criteria or competitive and negotiated procedures): not only in the European Member States due to the harmonization of EU Directives and Regulations, but also in other non-European countries such as United States, Canada, Australia or Latin America. Moreover, regarding oblivious indicators, they are mainly related to ordinary activities in awarding public works contracts. So, for instance, subcontracts, document verifications or worksite visits are typically provided not only in European countries (e.g., France, Germany or Spain), but also in non-European ones such as the United States or Canada.^21^ Thus, the relevance of these new indicators has the potential to be rather broad.

## Main and verification data: descriptive statistics

We verify the presence of our red flags in two different datasets: Main and Verification data. Our Main data contain all of the public tenders for roadwork jobs with a reserve price in excess of 40,000 euros and awarded by counties and municipalities between January 2009 and November 2015.^22^The Italian legislation requires to categorize public procurement contracts by the type of job involved: roadwork jobs are the most frequent job type, accounting for about a third of all contracts for public works awarded. We focus on roadwork contracts not only for their relevance, but also because they are relatively standardized as they typically involve simple tasks, mostly paving jobs and other maintenance works on roads, highways, and bridges. To ensure the comparability with our verification data, we focus on the procurements held in seven regions: three in the North (Lombardy, Piedmont, Veneto), two in Center (Lazio and Umbria) and two in the South (Campania and Sicily). The resulting dataset contains 12,786 contracts.

Our Verification data includes 3553 contracts for which we obtained both the call for tenders and award notice documentation. The call for tenders is the document with which the PA announces publicly that a tendering procedure is ongoing. At the same time, the award notice describes the outcomes of this procedure concerning the winning firm, the winning price, and, possibly, the list of other participants and their losing bids. These contracts involve the same period, type of jobs, and geographical regions of the contracts in the Main data. However, the two datasets originate from two different sources and cover a slightly different set of contracts: the Main data are from the Italian Anticorruption Authority (ANAC), which is the public body in charge of supervising the Italian public procurement system. The Verification data are from a private company (Telemat) that collects and resells to potential bidders detailed tender documentation. About 60% of the contracts in the Verification are also part of the Main data. The remaining ones do not clear the 40,000 reserve price threshold at which the ANAC data recording starts.

A. *Descriptive Evidence on the two datasets*. Table 2 presents summary statistics separately for the two datasets. In panel A, we present some basic tender characteristics, as the reserve price (i.e., the publicly announced maximum price the PA is willing to pay), the winning discount (the bid’s rebate over the reserve price) and the number of bidders (both overall and for the subset of bids clearing admissibility checks; the last row reports the number of invited bidders, as some tenders are by invitation only). Comparing the statistics for the two sets of data reveals several differences. The Verification data contracts have a reserve price that is both higher on average and substantially more dispersed. They also have a higher number of bidders, both invited and effective.

**Table 2:** Summary statistics for the main and verification data

	Main data			Verification data		
	Mean	S.D.	N	Mean	S.D.	N
A. *Basic tender characteristics*						
Reserve Price (000)	266.38	370.87	12,786	455.17	718.55	3200
Winner Discount	18.80	13.54	12,500	23.07	12.96	3439
No. Bidders	17.27	41.67	12,822	46.77	65.34	3486
No. Accepted Bidders	16.20	39.31	12,822	44.52	62.01	3486
No. Invited Bidders	5.14	12.68	12,822	14.18	21.97	1089
B. *Accessible indicators*						
Design-Build	0.00		12,823	0.02		3155
Urgency	0.02		12,823	0.01		2812
Negotiated	0.78		12,814	0.54		2697
Negotiated-No Tender	0.96		10,010	0.32		1454
Price Only—w. ABA	0.26		9780	0.63		3154
Scoring Rule (MEAT)	0.10		9753	0.08		3314
Open Tender Days	22.07	11.54	12,420	32.06	15.60	2436
Open Tender Day V.	0.39		12,420	0.11		2420
C. *Oblivious indicators*						
Tender Call Absence				0.33		3553
Page Count				25.54	16.55	2384
Word Count (000)				9.17	8.90	2384
Legality Protocols				0.32		2402
Local Regulations				0.33		2449
Negotiated-No T/N				0.23		1454
Sole Source Forbidden				0.03		2408
Average Qualit. Score				3.84	15.92	3553
Firm List Preference				0.04		2397
Firm Other Preference				0.28		2396
Documents Verificat				0.54		2392
Worksite Verificat				0.51		2392
Ad Hoc Subcontract				0.21		2391
No Subcontr to Bid				0.21		2391
Contact Points Out				0.30		1439
DV-Hours Share				0.77	0.32	2682
WV-Hours Share				0.95	0.19	2687

Note: refer to Appendix A for an enhanced description of each variable. The table does not report the standard deviation for dummy variables.

B. *Descriptive evidence on the indicators*. In panels B and C, we compare the two datasets along with our red flags. As mentioned in Sect. 3, we refer to the set of variables in panel B as the Accessible Indicators because they can be readily computed and used by the Anticorruption Authority, which maintains the sector’s supervision through the dataset from which the Main data have been extracted. In panel C, instead, we report additional characteristics that are not currently part of the data collection effort of the Anticorruption Authority,^23^ and refer to them as Oblivious Indicators.

Accessible indicators are available for both datasets (panel B) and oblivious indicators only for the Verification data, for which they were specially collected (panel C). As concerns partitioning by activity type, we emphasize that the indicators involving Information completeness display missing calls for tenders in 33% of the cases. In contrast, information which contracting authorities have to communicate to ANAC is not available for 42% of procedures. On average, calls for tender are 25 pages long and contain about 9000 words. Concerning the red flags related to the category of awarding procedures, we note that the cases in which PAs use negotiated procedures under specific conditions of urgency surprisingly represent only 1% of our sample. In the remaining cases (41% of our sample), they are the result of a discretionary choice of the contracting authority. Negotiated procedures without the publication of a call for tenders represent 13% of our sample, while negotiated procedures without the publication of any other notice are 9%. Legality protocols apply in 32% of cases, while local regulations are present in 33%. The design-build project delivery method accounts for 2% of contracts.

As regards awarding criteria indicators, most contracts (63%) are awarded using the lowest price criterion and the automatic exclusion of abnormal tenders (ABA). ABA are intended to eliminate offers that, relative to a benchmark value (often set to the average of the bids submitted) are deemed too risky to be acceptable as they might lead to future contract renegotiations. The MEAT criterion is used only in 8% of procedures: in these cases, the technical score incidence is predominant compared to the qualitative one. Single source awards are allowed in 97% of cases.

Finally, for indicators of obstacles to participation, their presence in the Verification data is summarized by the statistics in panel C. For the two preferred firm indications, a firm register is used in 4% of cases. In contrast, other indications of preferred firms are present in 28%. Given an average of 32 days to submit a tender (see open tender days in panel B), the instances in which the number of days provided by the call for tenders is less than the minimum required by law occur in 11% of the cases. Document verification is mandatory in 54% of procedures, worksite verification in 51%. Ad hoc rules for subcontracting are present in 42% of cases: 21% of calls for tender provide for a clause which prohibits the use of subcontracting, while 21% establish rules beyond those provided by law. Multiple contact points for economic operators are present in 5% of cases, external contact points in 30%.

## Indicators’ validation

The literature and operating practices provide no shortage of red flags. However, which of them are truly important to detect corruption? There is little systematic evidence to answer this fundamental question, and the reason lies in the scarcity of reliable outcome measures of corruption. Both direct measures of judicial cases and indirect measures involving the price/quality ratio of what procured face the problems discussed earlier.

The second contribution of this paper is that of validating the proposed indicators. We do so through new measures of firm-level corruption risk. In particular, our primary measure comes from Decarolis et al. [22], and it is based on police investigations: it allows us to observe for each firm winning a contract an indicator of whether any of its owners or top managers have been the object of a police investigation for corruption-related crimes.^24^ More precisely, the three types of crimes considered are (i) corruption, malfeasance, and embezzlement, (ii) abuse of power and undue influence, and (iii) violations in public auctions. The indicator variable, *criminal*, thus takes the value of one whenever the firm has at least one of its owners or top managers ever investigated by any Italian police force (civil or military) for at least one crime of the types mentioned above. The usage of an indicator variable rather than the number of crimes (or crimes per person) limits the danger of merely capturing a proxy for firms’ size. Although the opening of an investigation is by no means a proof of corruption, given the difficulty of capturing the phenomenon of interest, we consider this approach as appropriate to identify firms that are at risk. Furthermore, as explained in greater detail in Decarolis et al. [22], the typical flagged firm in our record implies another firm making allegations of corruption, the police conducting for roughly a couple of weeks preliminary investigations to assess the reasonableness of these allegations and, only then, formally opening the investigation that is at the basis of our measure. Thus, while false positive are undoubtedly present, our measure is not a mere list of allegations.

In Table 3, we report summary statistics for both datasets for five different corruption outcome measures. Our main measure, *Criminal*, appears in the first row, followed by four alternative corruption measures: two based on direct judicial evidence (*convicted* and *debarred*) or indirect economic outcomes (*extra cost* and *extra time*). The incidence of *criminal* for firms across the two datasets is very consistent: 15% of contracts are won by corruption risk firms.^25^

**Table 3:** Summary statistics: outcomes

	Main data			Verification data		
	Mean	S.D.	N	Mean	S.D.	N
Criminal	0.15	0.36	11,752	0.15	0.36	3195
Convicted	0.02	0.12	11,752	0.01	0.11	3195
Debarred	0.01	0.09	11,752	0.01	0.12	3195
Extra Cost	0.07	0.15	5122	0.10	0.17	715
Extra Time	0.65	0.77	3576	0.48	0.68	703

Not surprisingly, the extent of corruption appears much smaller and nearly negligible if measured through judicial data. In particular, to build the *convicted* measure, we reviewed all of the conviction sentences for corruption cases involving public procurement by the highest court (*Corte di Cassazione*) in the period 1995–2015. We then traced back the whole set of firms involved in the case (by reviewing the first two degrees of judgment preceding the one in front of the highest court). However, when matched with our datasets, only 2% of the contracts won in the Main data, and 1% of those in the Verification data are awarded to convicted firms. This fact confirms the limited possibility of using judicial data as a measure of corruption (see paragraph 2). It is in line with what legal scholars and policymakers have lamented about the Italian legal framework to combat corruption, which appears incapable of using convictions as a deterrent.^26^

The other judiciary measure, *debarred*, measures a peculiar tool meant to combat criminal (especially mafia) infiltrations in public contracts. Even without conviction sentences, firms can be excluded (i.e., debarred) from the awarding of public contracts if the local police forces signal that, based on the available evidence, they present serious risks of criminal infiltration. Nevertheless, this measure can be appealed in court, which is why we consider it a judicial measure. In our data, the instances of contracts awarded to firms that were ever subject to at least one debarment are minimal with just 1% of the cases.^27^

While the judicial measures suffer from underestimating the corruption phenomenon, the two alternative measures that suffer from measuring it very imprecisely are based on economic outcomes. In both datasets, the average contract experiences a substantial delay in its execution: an *extra time* of 50%, which is about the average, indicates that the execution of the work took one time and half what was initially established at the time of the contract awarding. The cost overruns measured by *extra cost* are also not negligible, albeit less striking. However, there are two main problems with these variables. First, the data are incomplete and, most likely, selected: in both datasets, the information is available for less than half of the contracts. Second, even if the data were complete, it would not be immediate to associate poor contract performance and corruption. As Bandiera, Prat and Valletti [5] showed for the procurement of standardized goods, the presence of bureaucratic inefficiency might lead to overestimating corruption. Furthermore, for the procurement of public work, as renegotiation might be an optimal strategy for complex contracts (Herweg and Schwarz [35]).

Table 4 offers clear evidence on the limits of using *extra cost* and *extra time* as indirect corruption outcomes. This matrix of correlations among the five outcome measures shows that both measures are practically uncorrelated with both *criminal* and the two judicial variables, both *debarred* and *convicted*. Indeed, despite the statistically significant correlation between the latter two variables and *extra cost*, the magnitude of the correlation coefficient is negligible. The same observation, however, also applies in the case of the correlation between *criminal* and the two judicial variables, which is not surprising given the minimal variation of these two variables observed in the data. Overall, we consider the evidence as strongly indicative of the greater merits of *criminal* as a measure of corruption than the four other alternative measures collected. This conclusion is also supported when we disaggregate the data at the regional level, as reported in Table 5. The rest of the analysis thus uses *criminal* as the outcome of the regression and prediction models presented next.^28^

**Table 4:** Outcomes correlation matrix

	Criminal	Convicted	Debarred	Extra cost	Extra time
Criminal	1				
Convicted	0.107^∗∗∗^	1			
Debarred	0.053^∗∗∗^	−0.001	1		
Extra Cost	0.001	0.047^∗∗∗^	0.035^∗∗^	1	
Extra Time	−0.013	−0.017	−0.009	0.095^∗∗∗^	1

^∗^ *p*<0.05, ^∗∗^ *p*<0.01, ^∗∗∗^ *p*<0.001.

**Table 5:** Summary statistics: outcomes, by region

Regione	Criminal	Convicted	Debarred	Extra cost	Extra time
Campania	0.14	0.00	0.03	0.12	0.78
Lazio	0.20	0.01	0.03	0.06	0.29
Lombardia	0.19	0.01	0.01	0.11	0.65
Piemonte	0.22	0.06	0.01	0.12	0.53
Sicilia	0.17	0.003	0.04	0.10	0.52
Umbria	0.21	0.00	0.00	0.37	0.73
Veneto	0.08	0.00	0.00	0.09	0.59

## Empirical strategy

The objective of our empirical analysis is to determine whether red flags help to predict corruption. Hence, we are not seeking the estimation of the causal effect of one (or more) of these indicators. However, we are interested in how red flags obtainable from tender notices serve to correctly predict that a contract is awarded to a corruption risk firm. This interest in model selection is more typical of the ML literature than of economics. Nevertheless, we see our problem as one of those economic questions considered well suited for ML methods.^29^ This is for at least three reasons.

First, in the typical economic study, model selection happens through knowledge of the market forces. Here, all indicators are, at least in principle, entirely plausible based on existing theories or operating practices. This underscores the elusiveness of the corruption problem that we analyze. Our goal is not to test one (or more) of these theories and heuristics, but to let the data drive the model selection stage. Indeed, a novelty of our contribution is precisely to propose new indicators and to validate them.^30^ Second, several of the proposed indicators cannot have a ground truth *causal* role, but are nevertheless interesting from a policy perspective. For instance, take the case of the number of days a call for tenders is open for bidding: finding that shorter periods are associated with more corruption is policy-relevant if corruption is societally costly and decisions have to be made on what contracts to investigate. However, it is unlikely that corruption is *caused* by shorter bidding periods. Allowing bureaucrats discretion over the length of this period can facilitate corruption, but it is unlikely to be a profound driver of the phenomenon.^31^ Third, the ML emphasis on model fit is particularly appropriate given the nature of the oblivious indicators. It offers a way to assess the usefulness of investing in learning these indicators beyond our Verification data.

Therefore, in the spirit of Kleinberg et al. [42], our strategy to use ML tools in economics entails using off-the-shelf ML methods. Within the vast and growing ML literature, our analysis lives within the context of “supervised learning” and, hence, we focus on three workhorse algorithms: LASSO, Ridge regression and random forests.^32^ The first two are regularization methods aimed at reducing the dimensionality of the model specification, by either dropping (LASSO) or shrinking (Ridge) some of the covariates. Both algorithms are well known in economics, being in several ways the tools in ML closest to an OLS.^33^ But contrary to an OLS, these methods are algorithms requiring the user to make some choices when applying them to the data at hand. This issue is even more pronounced with random forests. Although this algorithm inherits the simplicity and intuitiveness of the tree-based classification approaches, it also requires some adaptations.^34^ To minimize the arbitrariness of our choices and ensure replicability, we implement all three algorithms through commonly used statistical packages.^35^

Our data structure shows both shared and different features from the typical ML exercise. As typical in ML, we have a large number of potential predictors, while observing a relatively small set of contracts. In this context, we acknowledge standard techniques such as OLS are known to perform poorly and to be inferior to alternatives proposed by the ML literature. In this sense, looking at LASSO and Ridge regression is a natural starting point as the development of these methods originated to address this type of problem. Nevertheless, the results below will also clearly point toward the usefulness of the random forest algorithm. This is likely due to its more significant functional form flexibility, combined with the dense nature of our data. For all methods, we will report measures of their prediction accuracy. However, where our data departs from the typical ML setting. We acknowledge that our two datasets have potentially different distributions of the relevant variables. Hence, our two samples shall not be confused with the training and validation data to which the ML literature refers. Furthermore, while we observe the outcome variable in both datasets, it is the set of indicators that differs. Verification data can be analyzed through either a large model with all indicators or a small one using only a subset of them. Nevertheless, only the latter, small model is feasible for the Main data. Our interest is in learning how this difference limits the ability of a few standard algorithms to predict the outcome accurately.

## Results

We begin the presentation of the results by contrasting OLS estimates with those obtained with the two ML workhorse algorithms: LASSO and Ridge regression. After discussing the results for both the Main and Verification data, we introduce the findings from the random forests and, then, conclude by comparing all four methods to evaluate the contribution of the oblivious indicators.

A. *Main data findings*. The three columns of Table 6 report the estimated coefficients obtained through OLS, LASSO, and Ridge regression. The model specification includes all the indicators available in the Main data, as well as year, region, and reserve price range fixed effects. As this is a small set of indicators, the curse of dimensionality problem is unlikely to bite. However, for consistency with the analysis that follows, we apply ML algorithms with this restricted set of indicators. These indicators are those directly observed by the Anticorruption Authority, and, concerning the classification by type of activity involved, it entails mostly indicators of the awarding procedures and criteria groups. The OLS estimates indicate that the model has low explanatory power with an adjusted $\mathrm{R}^{2}$ of less than 4% and an MSE of 0.35. Among the individual coefficients, the only one that is statistically significant is that on the MEAT criterion. Contracts awarded using this multi-criteria approach are positively associated with corruption risk winners. This indication is in line with what the theory would suggest, and it is an interesting finding given the widespread usage of this type of criterion.^36^

**Table 6:** Estimates for the small model—main data

	OLS	LASSO	Ridge
	Corruption risk	Corruption risk	Corruption risk
Design-Build	0.003	0.002	0.003
	[0.004]		
Urgency	−0.005	−0.003	−0.004
	[0.003]		
Negotiated	0.005	0.000	0.002
	[0.005]		
Negotiated-No Tender	0.003	0.001	0.003
	[0.004]		
Price Only—w. ABA	−0.003	−0.003	−0.003
	[0.004]		
Scoring Rule (MEAT)	0.008^∗^	0.008	0.008
	[0.004]		
Open Tender Days	−0.003	0.000	−0.001
	[0.005]		
Open Tender Day V.	0.000	0.001	0.001
	[0.004]		
Observations	12,623	12,623	12,623
Adj R2	0.036		
MSE	0.355	0.126	0.126
False Positive	3323	3352	3334
False Negative	2159	2158	2170
Precision	0.228	0.227	0.225
Recall	0.312	0.313	0.309
F Measure	0.264	0.263	0.261
Threshold	0.192	0.189	0.189

^∗^ *p*<0.1, ^∗∗^ *p*<0.05, ^∗∗∗^ *p*<0.01. All specifications include year and region fixed effects. Robust standard errors in parentheses for OLS estimates. Due to the limited number of observations in our sample, LASSO and Ridge regressions are evaluated through a standard k-fold cross-validation method (with *k* = 10), and not through the more common train-test split. MSE is equal to the root mean squared error for OLS, and to the minimal cross-validation mean squared error for LASSO and Ridge regressions. False Positive indicates the number of cases in which a non-corrupt firm is classified as corrupt by the model. False Negative indicates the number of cases in which a corrupt firm is classified as non-corrupt by the model. Threshold indicates the predicted value of the outcome variable for which a firm is classified a corrupt. See Appendix C for additional results including precision and recall graphs, and ROC curves.

LASSO and Ridge regressions confirm that the MEAT is the indicator with the highest magnitude coefficient for both algorithms. In line with the literature, we ensure the comparability of all the coefficients across the three columns by centering and standardizing both outcomes and covariates. Thus, all three methods indicate a magnitude of the MEAT coefficient that is about twice as that of the next best indicator. The three methods also agree on what this second-best indicator is: urgency. Contrary to a naive view that the greater flexibility allowed to bureaucrats when they award contracts under the faster procedures allowed by invoking an urgency, urgency is negatively associated with corruption risk winners. The open tender days indicator shows a similar surprising negative effect. However, both indicators are possibly high on the list of the usual suspect for corrupt behavior of the Anticorruption Authority so that this evidence is compatible with actions aimed at avoiding detection by the monitoring entity.^37^ The sign on ABA is negative as well. Nevertheless, this finding is in line with our expectations: such an indicator marks contracts awarded with a lottery-style mechanism that is prone to bidders’ coordination and collusion, but very hard to pilot for a corrupt bureaucrat. All other indicators are positively associated with corruption risk winners, in line with the literature: design-and-build contracts (as opposed to build-only contracts), negotiated procedures (and negotiated without prior publication of the call for tenders), and violations in the minimum number of days during which the call for tenders is published. The LASSO model now drops two coefficients the Negotiated and the Open Tender days coefficients, whose magnitude the Ridge model indicates to be small too.

While the estimates reported in the three columns are remarkably similar, the models’ overall performance is disappointing. Although the MSE is halved in the ML methods if compared to the OLS one, the prediction is highly inaccurate for all the three methods. This is showed by the high fraction of both type I error (false positive) and type II error (false negative) reported at the bottom of the table.^38^ For all three models, the former is about 26% of the cases, while the latter accounts for 17% of the cases. Random forests will allow substantial improvements in this classification accuracy, but before discussing that, we briefly examine the Verification data’s findings.

B. *Verification data findings*. We report estimates for the Verification data in Table 7. The algorithms are the same discussed above, but now we feed the algorithms with two different sets of indicators. For each algorithm, the first column considers a small model with the same set of indicators used for the Main data. The second column uses a large model that includes all available indicators. Furthermore, for the large model, we also include 20 dummy variables to account for all instances in which some of the contracts have indicators that cannot be (unambiguously) assessed.^39^

**Table 7:** Estimates for the small and large models—verification data

	OLS		LASSO		Ridge	
	Corruption risk	Corruption risk	Corruption risk	Corruption risk	Corruption risk	Corruption risk
Design-Build	−0.008	−0.009	0.000	0.000	−0.001	−0.000
	[0.008]	[0.008]				
Urgency	−0.005	−0.004	−0.003	−0.001	−0.003	−0.002
	[0.005]	[0.005]				
Negotiated	−0.003	−0.000	0.000	0.000	0.000	0.000
	[0.013]	[0.013]				
Negotiated-No Tender	0.007	0.008	0.004	0.000	0.004	0.002
	[0.009]	[0.013]				
Price Only—w. ABA	0.012	0.010	0.000	0.000	−0.004	−0.005
	[0.009]	[0.009]				
Scoring Rule (MEAT)	0.011	−0.005	0.008	0.000	0.008	0.003
	[0.009]	[0.013]				
Open Tender Days	0.009	0.008	0.011	0.010	0.012	0.009
	[0.010]	[0.010]				
Open Tender Day V.	0.021^∗∗^	0.021^∗∗^	0.020	0.016	0.017	0.012
	[0.008]	[0.008]				
Missing Tender Call		−0.076		0.000		−0.002
		[0.047]				
Page Count		0.002		0.000		0.002
		[0.009]				
Word Count		0.009		0.008		0.007
		[0.008]				
Legality Protocols		−0.003		0.000		−0.002
		[0.011]				
Local Regulations		−0.015		−0.006		−0.006
		[0.011]				
Negotiated-No T/N		−0.003		0.000		0.001
		[0.014]				
Sole Source Forbidden		−0.010^∗^		−0.007		−0.006
		[0.005]				
MEAT-Qual. Score		0.024^∗^		0.015		0.010
		[0.013]				
Firm List Preference		0.007		0.003		0.003
		[0.007]				
Firm Other Preference		−0.005		0.000		−0.000
		[0.008]				
Documents Verificat		−0.008		−0.005		−0.002
		[0.017]				
Worksite Verificat		0.011		0.000		0.000
		[0.015]				
Ad Hoc Subcontract		−0.067^∗∗∗^		−0.004		−0.004
		[0.012]				
No Subcontr to Bid		0.068^∗∗∗^		0.000		−0.001
		[0.014]				
Contact Points Out		0.007		0.002		0.004
		[0.009]				
DV-Hours Share		0.008		0.005		0.005
		[0.011]				
WV-Hours Share		0.012		0.009		0.008
		[0.007]				
Observations	3195	3195	3195	3195	3195	3195
Adj R2	0.028	0.029				
MSE	0.356	0.356	0.129	0.128	0.128	0.128
False Positive	649	643	699	696	750	730
False Negative	630	623	625	628	622	620
Precision	0.255	0.257	0.245	0.246	0.235	0.240
Recall	0.261	0.263	0.266	0.265	0.270	0.271
F Measure	0.258	0.260	0.255	0.255	0.251	0.254
Threshold	0.196	0.201	0.176	0.177	0.175	0.175

^∗^ *p*<0.1, ^∗∗^ *p*<0.05, ^∗∗∗^ *p*<0.01. All specifications include year and region fixed effects. All the choices involving standard error, validation, and MSE calculations are identical to those reported in the note to Table 6.

There are several impressive results on the individual indicators that we can learn from the large model. First, the association between the MEAT criterion and corruption risk is stronger the more the scoring rule assigns points to qualitative (as opposed to quantitative) parameters. In contrast to the previous findings, all three methods indicate that the violation in the minimum number of days for which the call for tenders is open is a significant predictor. The large model also allows us to discover the relevance of several indicators, mostly belonging to the group that we classified as obstacles to participation. In particular, we observe the importance of some features related to the existence and the characteristics of the obligations involving both access to the tender documentation and the worksite inspection. The easier it is satisfying these requirements (in terms of allowing a larger share of time during the bidding period in which these obligations can be satisfied), the less likely the winner is a corruption risk firm. The estimates in the Table reveal that the broad set of indicators include some indicators positively linked with corruption and others negatively associated to it. There are also indicators that the LASSO completely drops and that the Ridge regression shrinks to nearly zero. Most of the indicators covering information completeness are of this kind, with the only exception of the number of words in the call for tenders, whose coefficient is however rather small in magnitude.

In terms of the overall model fit, the low predictive ability discussed earlier for the Main data also applies to the Verification data. Interestingly, the appropriate measure improves little when moving from the small to the large model. The findings are quantitatively very close to those reported for the Main data along with the three dimensions of MSE and type I and II errors.

To improve on these measures, we then introduce a random forest approach. The random forest algorithm provides us with a more accurate estimate of the error rate compared with standard decision trees (Breiman [10]). The out-of-bag error during the training process measures the error rate. In each tree of the random forest, the out-of-bag error is calculated based on predictions for observations not in the bootstrap sub-sample. After training the random forest algorithm, it is possible to get estimates of the relative importance of each of the covariates in terms of predictive power.^40^

**Figure 1:**
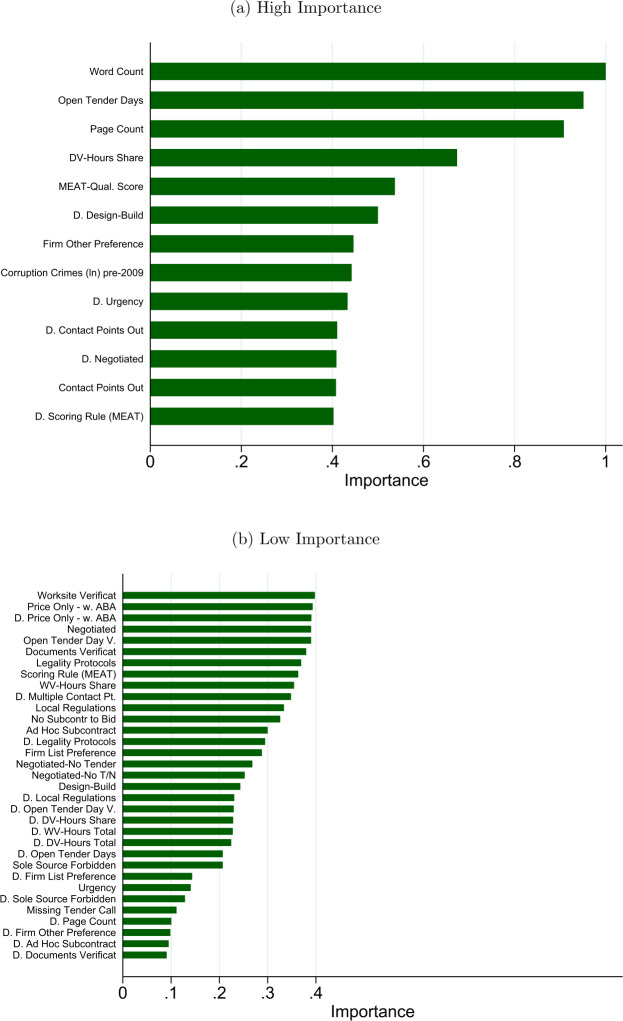
Random forest

Contrary to regression models, there is no simple way to represent the results of a random forest fully. Figure 1 reports the importance of the indicators, separating high (top panel) and low (bottom panel) importance indicators. This type of visualization describes how much each indicator contributes to the average decrease in impurity over trees. Although routinely used to summarize, for a given model, those features most important in explaining the target variable have well-known biases. Therefore, we comment on it only briefly and then move on to the discussion of the entire model in terms of the MSE.

In Fig. 1, we immediately see that the random forest agrees with the other methods concerning some variables (such as on the importance of the MEAT awarding criterion), but not for all. Indeed, we find that many of the information completeness indicators are highly relevant according to random forest’s prediction: the total number of both words and pages in the call for tenders are among the top red flags in terms of importance, while being only marginally relevant (albeit never fully excluded) under the alternative prediction models. Through Fig. 1, we can also offer a visual representation of the various additional controls—fixed effects and dummy variables for missing red flag data—that were included in the earlier models, but not reported for readability.

Interestingly, indicators for those regions often considered more at risk of corruption do not show up prominently. Also relevant is that the indicators for missing records are mostly concentrated—albeit with some exceptions—among the lesser relevant indicators, thus suggesting that the incompleteness of the call for tenders is not a significant driver in the findings. However, this is also in part attributable to the type of results representation used, which over-represents the importance of continuous features and high-cardinality categorical variables.^41^ We now turn to a comparison of the models to better assess the usefulness of the random forest model relative to the other models, especially concerning the inclusion of the oblivious indicators.

C. *Oblivious indicators contribution*. In Table 8, we report the prediction accuracy for all four methods across all datasets and models. The results for OLS, LASSO, and Ridge regression are those discussed above, and they are reported here only to ease their comparison with the random forest ones. Beginning with the top panel where we report results for the Verification data, the random forest appears to perform well in terms of both Precision and Recall measures, as its classification errors are substantially lower than the OLS, LASSO and Ridge models. This is expected since the random forests algorithm tends to fit very well in sample. However, passing from the Verification data to the substantially larger Main data leads to a worsening of the random forest due to an increase in the False Negatives, and so in the Recall and F Measure.

**Table 8:** Predictive accuracy across samples and models

Model	Small model			Large model		
	Precision	Recall	F measure	Precision	Recall	F measure
A. *Verification data*						
OLS	0.255	0.261	0.258	0.257	0.263	0.260
Lasso	0.245	0.266	0.255	0.246	0.265	0.255
Ridge	0.235	0.270	0.251	0.240	0.271	0.254
Random Forest	0.971	0.808	0.882	1	0.911	0.953
B. *Main data*						
OLS	0.228	0.312	0.264			
Lasso	0.227	0.313	0.263			
Ridge	0.225	0.309	0.261			
Random Forest	0.876	0.453	0.597			

Note: The measures presented in the table are calculated as follows: Precision = *Tp*/(*Tp* + *Fp*), Recall = *Tp*/(*Tp* + *Fn*), *F* = 2⋅Precision⋅Recall/(Precision + Recall). Where *Tp* indicates the true positives, *Fn* indicates the false negatives, and *Fp* indicates the false positives.

Finally, it is interesting to discuss how the different models respond to the inclusion of the large model’s oblivious indicators. The random forest model is the one whose performance in terms of F measure improves the most, while also retaining the lowest classification errors: passing from the small to the large model increases the F measure by 0.071, or 8% at its baseline value in the small sample. This model, likely due to the greater flexibility of its functional form, is better able to exploit the additional information provided by the inclusion of more red flags. Importantly, however, as the ranking of indicators in Fig. 1 shows, adding indicators is not only crucial because some of them are individually relevant, but for their overall contribution to the model performance.

D. *Causality and External Validity*. In terms of our approach in this study, the choice to adopt an ML approach is well-targeted because only some indicators have the potential to have causal effects.^42^ Some of the indicators are mere tools to achieve corruption and likely to be highly fungible with other tools. However, it would be of interest to explore the causal effects of those indicators that have the potential of ground truth causal effects: this would both enhance the interpretation of the earlier findings and increase the external validity of our findings.

Although reliably assessing the causal nature of the estimates discussed above is beyond the scope of this work, there are two considerations worth making. First, additional results presented in Table A.3 of the Appendix show the robustness of our baseline findings to alternative formulation of the LASSO problem that, in our context, evaluate the true effect of a subset of red flags on the outcome of interest (i.e., criminal firm winning). Knowing this effect in the true underlying model that generated the data being analyzed is hard because of the role of the other determinants of the outcome. The three models presented in the Appendix are increasingly reliable and require increasing computational power and time to be run, they are: Belloni, Chernozhukov and Hansen’s [7] double selection algorithm, Belloni et al.’s [6] partialing-out algorithm, and Chernozhukov et al.’s [18] cross fit partialing out. In essence, for the subset of red flags that the findings above indicated to be of particular interest, we apply these methods which are meant to allow conducting valid inference on these parameters, while controlling in flexible ways for all other confounding factors. Their validity requires an assumption of unconfoundedness, by which it is meant that no omitted variable bias is plaguing the analysis. Since such an assumption is hard to maintain in our setting, these results are only reported as a robustness in an Appendix. However, it is reassuring that the estimates in Table A.3 confirm the baseline results.

The second consideration about causation regards the fact that, given the specific task at hand there are specific problems related to the causal interpretation of parameters. For instance, is a natural concern that some of the red flags might become ‘invalidated’ for future works (either academic or investigative) once their role as effective corruption indicators is made public. Clearly, there is a concern of this kind only if enough agents in the market are induced to modify their behaviour by the belief that investigators have started to monitor certain red flags. In the short run, this seems unlikely that our work can have this effect as our analysis builds on pre-existing academic works and operating practices: if investigators were careful and proactive, they would have already monitored the relevant red flags that we discuss. It thus seem unlikely that corrupt agents could see our study as a game changer giving to investigators such increased detection capabilities as to require a change of behavior. This is especially true for those behavioral changes that are costly as, for instance, they entail less effective ways to steer the contract award to a favourite firm. In the long run, there is an always ongoing game of cat-and-mouse with corrupt agents adapting their behavior to deceive investigators. Our results might contribute to fuel this continuous process, but they would do so together with many other propellants, like past investigations, court cases, journal articles on mass media, etc.

Despite this ever evolving situation, it is important to stress why the substantive findings on the red flags reported in the paper are not worthless. In particular, we would like to argue that the extent to which the external validity of a red flag is reduced is both the motive that you mention (i.e., adaptation by strategic agents) and its specificity to the legal, institutional and technological environment. But both concerns are highly heterogenous across indicators. For instance, adaptation behaviour is less likely the more it is costly. Consider as an example a corrupt public buyer faced with the choice of running a price-only auction or a MEAT auction, and assume (as it is the case) that the latter can be more easily steered toward a favoured bidder. Then, a corrupt agent would adapt and abandon MEAT in favor of price-only, exclusively if the perceived increase in the probability of getting caught when using a MEAT is sufficiently high relatively to when a price-only auction is used. Thus, the fact that MEAT might entail more attentive monitoring by investigators looking for corruption does not imply by itself that the corrupt agent will not resort to MEAT. Similarly, regarding the second concern about specificity to the legal and institutional system, some of the features captured by the proposed red flags are ubiquitous in public procurement. Remaining with the same example as above, the choice between MEAT and price-only auctions is nearly always present across the public procurement regulations of different countries.

Finally, let us conclude this section by commenting on the possible external validity concerns. As discussed above, the issue of external validity might be not only across settings (as stated in your comment) but also within the same setting over time (if beliefs of agents about what investigators monitor evolve). However, as discussed, in our response above to your previous comment, for a subset of indicators, their external relevance is likely ensured by both their presence across many settings and the costs that changing behavior would impose on corrupt agents. Indeed, if we consider the four conditions that experimental economists consider when judging the external validity of an experiment,^43^ our analysis performs well in terms of the naturalness of the setting, choices, tasks and time frame observed: the procurement regulations of Italy are shaped by the EU procurement directives which regulate procurement in the whole EU and, to a large extent, are similar to those of most western countries. We also have no concerns in terms of attrition rates and non compliance because our dataset are representative of the national procurement of roadwork contracts (the Main data from contain all public contracts, while the Verification data are a random sample out of the set of all contracts). Lastly, regarding representativeness of the sample with respect to the full population, it depends what one considers as the full population of interest. The Italian setting is large enough that it would be both economically and socially relevant by itself as the target of the analysis. However, we shall stress that Italy is heterogenous in terms of its corruption risk and many areas are low risk, to the point that major increases in the extent of discretion given to public officers in charge of contract award had no impacts on awards to criminal firms: this is a striking result in Decarolis et al. [22] who examine reforms expanding the cases in which buyers can use negotiated procedures. In the revision, we emphasize these aspects to argue why there are substantive elements to consider our analysis to be externally valid.

## Conclusions

In this study, we exploited new contract-level data that we directly collected from the call for tenders documents and through data warehouse of the public entity monitoring corruption risk in Italian public procurement. We use these data to measure a broad set of red flags for corruption, some novel to the economic literature, and part of the Italian judiciary’s operating practices. We then combined these red flags with detailed firm-level corruption measures allowing us to obtain a measure of corruption superior to most of the alternatives in the literature. Finally, using ML tools, we explored the usefulness of the red flags to predict contract-level corruption.

We succeed in determining that some indicators have an evident predictive power by comparing different methods and samples. We also show that, among ML methods, the random forests algorithm provides the most accurate prediction. More crucially, if this algorithm is used, considerable prediction improvements are attained by including those indicators that we directly collect but are not monitored by the supervising entity. Overall, these results constitute the first systematic evidence on the predictive contribution of a large number of red flags for corruption. Given the high perceived costs for society of corruption, our results offer a way to think about the benefits of investing in the collection of red flags for corruption, especially considering that many of these indicators can be standard across the public procurement sectors of many countries.^44^ Statistical tests are by no means a sufficient element for conviction, but can be fundamental to direct in the right direction the scarce resources of the monitoring authorities.^45^

From a policy point of view, our results highlight several relevant aspects. In addition to the more general points emphasized in the introduction, it is worth to mention that our findings indicate the need for a careful regulation of the more discretionary mechanisms for selecting private contractors (in particular, MEAT criterion and negotiated procedures). Discretion plays a crucial role in effective procurement, especially in the case of complex contracts. However, contracting authorities awarding contracts through the MEAT criterion should clearly define the objectives pursued in the call for tenders and prefer “measurable” parameters, that can be less easily manipulated. Furthermore, negotiating procedures show some advantages over competitive procedures, representing a faster and more flexible instrument for selecting private contractors. However, the provision of transparency requirements is essential, in particular, limiting the use of a negotiated procedure without the publication of any notice. Moreover, our analysis shows the relevance of monitoring compliance with the minimum time limit for submission of tenders and providing adequate controls on subcontracting, which represent an area vulnerable to corruption risks.

Finally, at a more general level, our analysis suggests that a higher standardization of call for tenders documents can contribute to reducing corruption risks. For this purpose, sector authorities or specialized public bodies can play a crucial role. In addition to diffusing best practices, these structures may contribute to harmonizing standards, increasing the degree of certainty of interpretation in a highly complex regulatory context. Moreover, an adequate centralization and professionalization of contracting authorities (*inter alia*, in terms of specialized technical skills and project management capability), should be ensured in order to select private contractors, also mitigating corruption risks properly.

## Figures and Tables

**Figure A.1:**
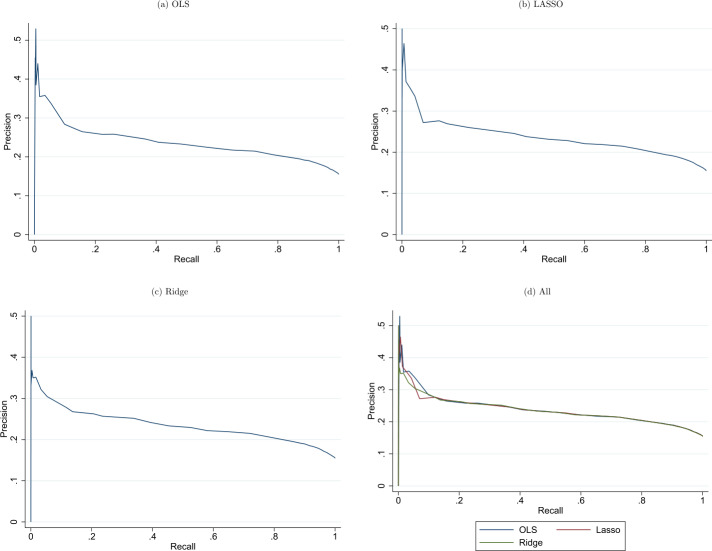
Precision and recall curves. Small model—main data

**Figure A.2:**
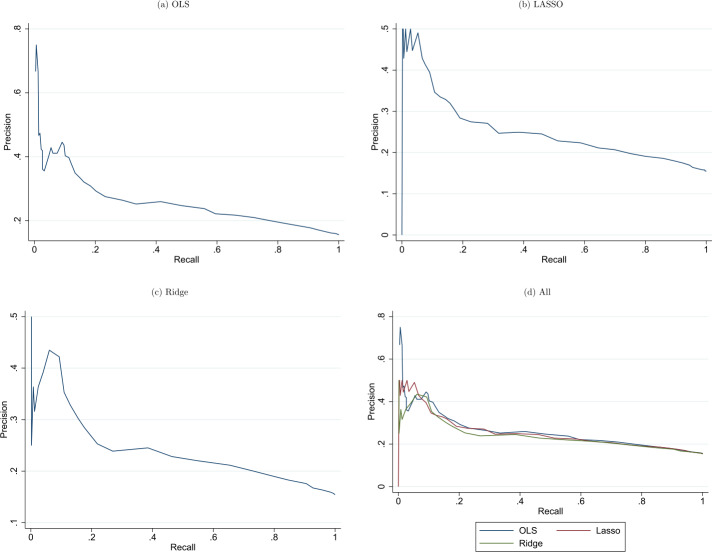
Precision and recall curves. Small model—verification data

**Figure A.3:**
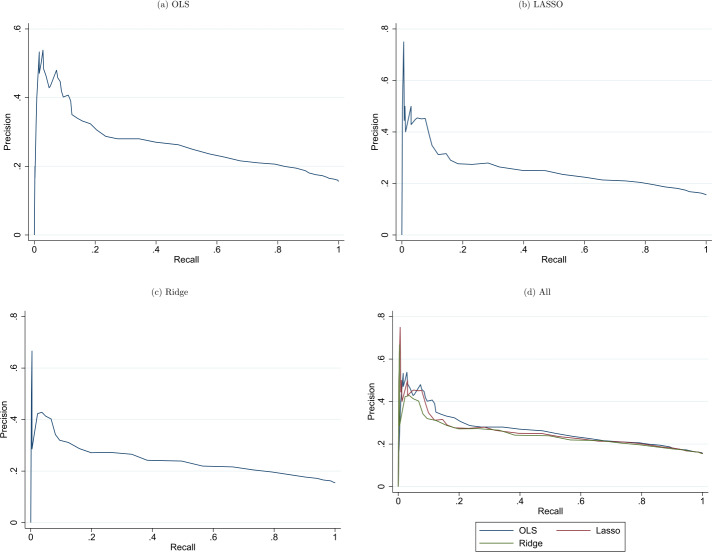
Precision and recall curves. Large model—verification data

**Figure A.4:**
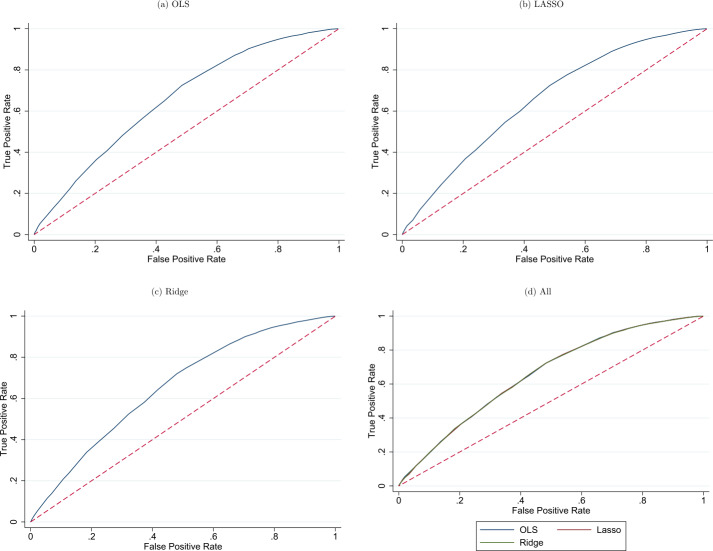
ROC curves. Small model—main data

**Figure A.5:**
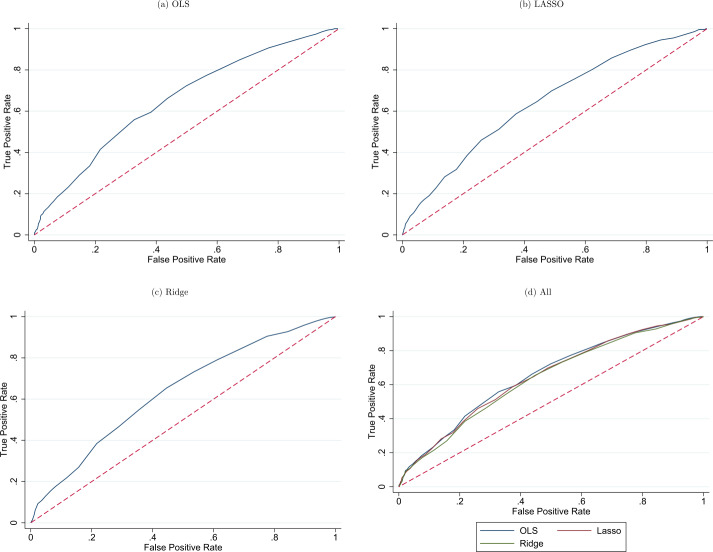
ROC curves. Small model—verification data

**Figure A.6:**
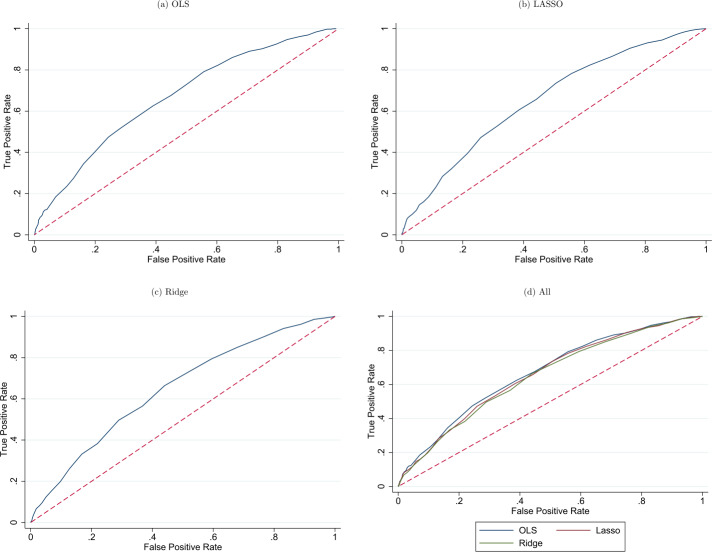
ROC curves. Large model—verification data

**Figure A.7:**
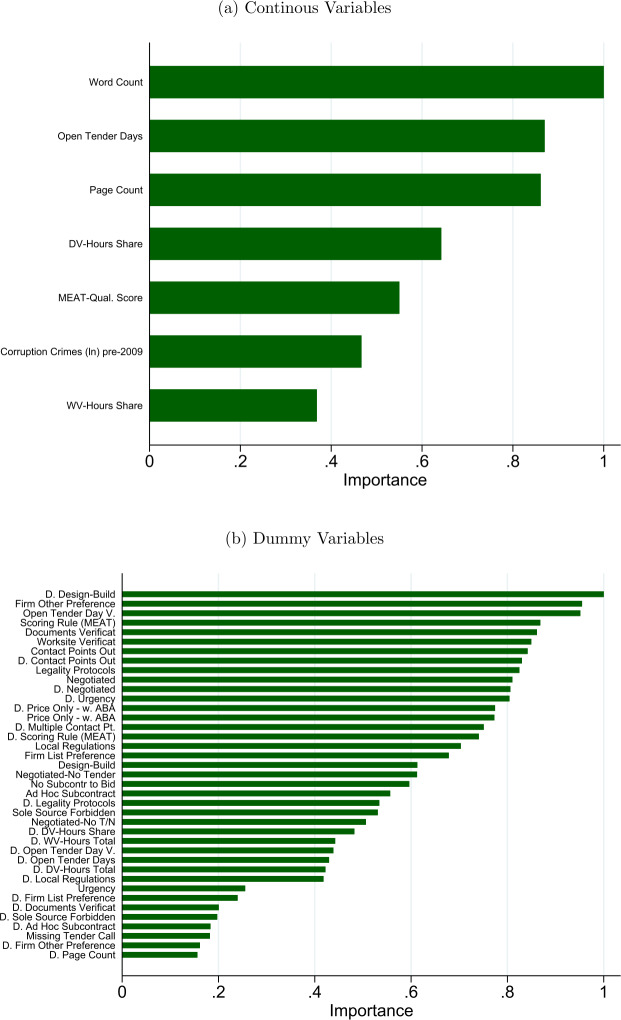
Random forest

## Data Availability

All the codes implementing the analysis are released in a replication package. The data cannot be shared in full because of confidentiality agreements detailed in Appendix B (Data Sources).

## Appendix A: Data details

In this section, we present a more detailed data description. Table A.1 lists all the variables used in the study and, for each of them, offers a brief description and indicates the data type.

**Table A.1:** Contract and tender data details

Variable	Description	Datatype
*Basic Tender Characteristics*		
Reserve Price	Maximum price that can be asked to the buyer	1000 Euro
Winner Discount	Percentage discount over the reserve price	Percentage
No. Bidders	Number of firms submitting a bid	Integer
No. Accepted Bidders	Number of firms whose bid is deemed compliant with the eligibility rules	Integer
No. Invited Bidders	Number of firms invited to submit a bid	Integer
Extra Cost	Difference between final and contract price, over the reserve price	Percentage
Extra Time	Difference between final and contract days-to-execute, over contract days-to-execute	Percentage
*Red Flags*		
Design-Build	Indicator for whether the contract involves both design and build (1) or only build (0)	Boolean
Urgency	Indicator for whether the call for tenders invokes any urgency clause (1) or not (0)	Boolean
Negotiated	Indicator for whether the procedure is negotiated (1) or not (0)	Boolean
Negotiated-No Tender	Indicator for whether the procedure is negotiated & no call publicity required (1) or not (0)	Boolean
Price Only—w. ABA	Indicator for price-only criterion & automatic exclusion of abnormally low bids (1) or not (0)	Boolean
Scoring Rule (MEAT)	Indicator for whether the award criterion entails multiple parameters (1) or not (0)	Boolean
Open Tender Days	Number of days between when the call is published and when it closes	Integer
Open Tender Day V.	Indicator for Open Tender Days below legal minimum (1) or not (0)	Boolean
Tender Call Absence	Indicator for whether the tender call is unavailable from Telemat (1) or not (0)	Boolean
Page Count	Number of pages of the call for tender main document	Integer
Word Count	Number of words (in thousands) of the call for tender main document	Integer
Legality Protocols	Indicator for whether the call request bidders to adhere to any legality protocol	Boolean
Local Regulations	Indicator for whether the call request bidders to adhere to any local regulation	Boolean
Negotiated-No T/N	For negotiated procedures, indicator for publicity of the call for tenders (1) or not (0)	Boolean
Sole Source Forbidden	Indicator for clauses forbidding to award with a single valid offer submitted (1) or not (0)	Boolean
Average Qualit. Score	For awards with MEAT, the average number of points assigned to quality parameters	Integer
Firm List Preference	Indicator for preferences for firms enrolled in the buyer’s preferred suppliers list (1) or not (0)	Boolean
Firm Other Preference	Indicator for preferences for specific firms, other than Firm List Preference, (1) or not (0)	Boolean
Documents Verificat	Indicator for compulsory verification of inspection of the project documents (1) or not (0)	Boolean
Worksite Verificat	Indicator for compulsory verification of inspection of the project worksite (1) or not (0)	Boolean
Ad Hoc Subcontract	Indicator for whether the call contains ad hoc rules for subcontracting (1) or not (0)	Boolean
No Subcontr to Bid	Indicator for whether the call forbids bidders to become subcontractors (1) or not (0)	Boolean
Contact Points Out	Indicator for contact point personnel outside the employees of the public buyer (1) or not (0)	Boolean
DV-Hours Share	Hours available for document verification over the total working hours during call opening	Percentage
WV-Hours Share	Hours available for worksite verification over the total working hours during call opening	Percentage

The first group of variables in the top panel of Table A.1 refers to tender features: *i*) reserve price is the price indicated in the call for tender as the maximum price that the buyer is willing to pay for the contract; *ii*) winner discount is the percentage discount over the reserve price that the winner gives to the buyer; *iii*) the latter three variables report, respectively, the number of bids presented, the number of valid bids presented and the number of invited bidders.^46^

The second group of variables in the top panel of Table A.1 refers to the red flags discussed in the paper. To ease their presentation, we describe them below according to their activity (see Table 1 in the main text).

The first category (information completeness) includes three indicators: *i*) the absence of a call for tenders; *ii*) the number of pages and words present in the call for tenders (when present); *iii*) the availability of information on the awarding and execution of the contract that contracting authorities have to communicate to the Anticorruption Authority (Autoritá Nazionale Anticorruzione—ANAC).^47^

Four indicators compose the category of awarding procedures: *i*) the use of negotiated procedures; *ii*) the application of legality protocols;^48^*iii*) the presence of local regulations;^49^*iv*) the use of design-build project delivery method.^50^ The first indicator includes four sub-indicators: *a*) use of a negotiated procedure; *b*) use of a negotiated procedure in urgent cases; *c*) use of a negotiated procedure without the publication of a call for tenders; *d*) use of a negotiated procedure without the publication of any other notice.

The category of awarding criteria includes three indicators: *i*) use of the most economically advantageous tender (MEAT) criterion; *ii*) the presence of a call for tenders clause which provides for the automatic exclusion of abnormal tenders (average bid auctions, ABA); *iii*) the presence of a call for tenders clause which does not allow a single-source award. The first indicator includes 3 sub-indicators: *a*) use of the MEAT criterion; *b*) incidence of the technical score (e.g., delivery time); *c*) incidence of the qualitative score (e.g., quality of work).

The last category (obstacles to participation) includes eight indicators: *i*) the presence of preferred firm indications; *ii*) the number of days to submit a tender from the date when the call for tenders was published (open tender days, OTD); *iii*) the presence of a call for tenders clause which provides for mandatory document verification (DV); *iv*) the presence of a call for tenders clause which provides for a mandatory worksite verification (WV); *v*) the presence of ad hoc rules for subcontracting; *vi*) the presence of a call for tenders clause which prohibits the use of pooling agreements (in the form of the so-called *avvalimento*); *vii*) the presence of multiple contact points for economic operators; viii) the presence of external contact points related to the contract. The first indicator (preferred firm indications) includes two sub-indicators: *a*) use of a firm register; *b*) other indications of preferred firms (e.g., specific requirements). The second indicator (OTD) involves two sub-indicators: *a*) the number of days to submit a tender from the date when the call for tenders was published; *b*) if this number of days is less than the minimum required by law. The third indicator (DV) includes four sub-indicators: *a*) the presence of a mandatory DV; *b*) provision of a specific date for the mandatory DV (DV Specific date); *c*) hour share for the mandatory DV of the total OTD (DV Hours Share); *d*) total hours for the mandatory DV (DV Hours total). The fourth indicator (WV) involves four sub-indicators: *a*) presence of a mandatory WV; *b*) provision of specific date for the mandatory WV (WV Specific date); *c*) hour share for the mandatory WV of the total OTD (WV Hours Share); *d*) total hours for the mandatory WV (WV Hours total). The fifth indicator (ad hoc rules for subcontracting) includes two sub-indicators: *a*) the presence of ad hoc rules for subcontracting; *b*) the presence of a call for tenders clause which prohibits the use of subcontracting.

## Appendix B: Data sources

The tender data used in the paper come from two main sources, corresponding to the two datasets used: the Main data and the Verification data. All contracts cover roadwork jobs: we selected this type of jobs because they are the most frequently procured types of public works and because they are relatively standardized as they typically involve simple tasks, mostly paving jobs and other maintenance works on roads, highways, and bridges. All contracts have been awarded between January 2009 and November 2015: this time window ensures that the contracts are sufficiently far away from two major legislative reforms of the Public Procurement Law of 2006 and 2016 that deeply affected the procurement system. Finally, we focus on contracts awarded by local administrations (counties and municipalities) located in seven regions: three in the North (Lombardy, Piedmont, Veneto), two in Center (Lazio and Umbria) and two in the South (Campania and Sicily).

Our Main data come from the Observatory on Public Contracts of the Italian Anticorruption Authority, https://www.anticorruzione.it/. The data contain all of the public tenders satisfying the criteria described in the previous paragraph and having a reserve price in excess of 40,000 euros (ANAC has a special monitoring system for contracts below this threshold). The resulting dataset contains 12,786 contracts. Although we accessed these data under an ad hoc research protocol, in the meantime the data have become publicly available through the web site: https://dati.anticorruzione.it/#/home. We shall remark that the data extracted from ANAC come from what this agency transmitted to us on October 2017. There is a constant influx of new data in the ANAC database, possibly overwriting past data (when corrections are deemed necessary or missing data are filled in). This issue, however, mostly affects contract execution dimensions (like *Extra Cost* and *Extra Time*) which play a very limited role in our analysis, as it is focused on features related to the call for tenders and the award notice.

The Verification data, instead, come from the direct inspection of the call for tenders of a random pool of contracts that we selected for this project. Out of all the contracts satisfying the criteria described in the opening paragraph of this section, we selected those for which we could access both the call for tenders and award notice documentation.^51^ We then randomly selected 3553 contracts and, through human reading of the documents, extracted the features described in Table A.1. Although we obtained the documents through a private company that collects, organizes and resells documentation on public contracts (http://www.telemat.it/),^52^ the documents are publicly available on the web site of each local administration.

For each of the features in Table A.1, we report in Table A.2 whether it is available only through the observation of the documentation that we obtained via Telemat or if it is collected by ANAC. Furthermore, for the tender documentation, we specify whether the feature is observable in the call for tenders or in the award notice.

**Table A.2:** Contract and tender data sources

Variable	ANAC	Tender documentation	Award notice
*Basic Tender Characteristics*			
Reserve Price	Yes	Yes	Sometimes
Winner Discount	Yes	No	Yes
No. Bidders	Yes	No	Yes
No. Accepted Bidders	Yes	No	Yes
No. Invited Bidders	Yes	No	Yes
Extra Cost	Yes	No	No
Extra Time	Yes	No	No
*Red Flags*			
Design-Build	Yes	Yes	No
Urgency	Yes	Yes	No
Negotiated	Yes	Yes	Sometimes
Negotiated-No Tender	Yes	Yes	Sometimes
Price Only—w. ABA	Yes	Yes	Yes
Scoring Rule (MEAT)	Yes	Yes	Yes
Open Tender Days	Yes	Yes	No
Open Tender Day V.	Yes	Yes	No
Tender Call Absence	No	Yes	No
Page Count	No	Yes	No
Word Count	No	Yes	No
Legality Protocols	No	Yes	No
Local Regulations	No	Yes	No
Negotiated-No T/N	No	Yes	No
Sole Source Forbidden	No	Yes	No
Average Qualit. Score	No	Yes	No
Firm List Preference	No	Yes	No
Firm Other Preference	No	Yes	No
Documents Verificat	No	Yes	No
Worksite Verificat	No	Yes	No
Ad Hoc Subcontract	No	Yes	No
No Subcontr to Bid	No	Yes	No
Contact Points Out	No	Yes	No
DV-Hours Share	No	Yes	No
WV-Hours Share	No	Yes	No

Finally, regarding the three judicial outcome features, they come from two sources. The indicator variables *criminal* and *debarred* come from the work of Decarolis et al. [22]. To use their data, we matched the unique identifiers of the winners in our data to theirs. They obtained this information for all firms ever involved in public works in the period between 2000 and 2017. The collection of such data was possible thanks to a framework agreement between the agency for the internal intelligence and security under the Presidency of the Council of Ministers and Bocconi University. An NDA agreement implies that we are not allowed to release this variable and, moreover, the need to satisfy this NDA constraints what we are allowed to present as a replication package in terms of datasets. To offer a useful replication package that does not violate this NDA, the package accompanying this paper generates at the beginning of the code two variables (as random draws from Binomial distributions) that are used as outcomes in place of the true *criminal* and *debarred* features. We have also removed the variable *corruption crimes pre-2009*, which is strictly confidential. All other variables in the dataset are the original ones.

Finally, specifically for this study, we built the *convicted* measure by reviewing all of the conviction sentences for corruption cases involving public procurement by the highest court (*Corte di Cassazione*) in the period 1995–2015.

## Appendix C: Additional results

This section reports additional results and performance measures. Table A.3 shows the results of the inferencial evaluation of the LASSO model through three different methods. The three models presented are increasingly reliable and require increasing computational power and time to be run. For more details on the models selected see: Belloni, Chernozhukov and Hansen [7] for the double selection algorithm, Belloni et al. [6] for the partialing-out algorithm, and Chernozhukov et al. [18] for cross fit partialing out.

**Table A.3:** LASSO inferencial results

	Double selection	Partialing-out	Cross-fit partialing-out
Open Tender Day V.	0.019^∗∗^	0.019^∗∗^	0.023^∗∗∗^
	[0.008]	[0.008]	[0.008]
Ad Hoc Subcontract	−0.059^∗∗∗^	−0.055^∗∗∗^	−0.054^∗∗∗^
	[0.011]	[0.007]	[0.012]
No Subcontr to Bid	0.060^∗∗∗^	0.052^∗∗∗^	0.052^∗∗∗^
	[0.013]	[0.009]	[0.013]
Firm List Preference	0.005	0.004	0.005
	[0.007]	[0.007]	[0.007]
MEAT-Qual. Score	0.024^∗^	0.023^∗^	0.022^∗^
	[0.012]	[0.012]	[0.012]
Observations	3195	3195	3195
Number of controls included	29	29	33

^∗^ *p*<0.1, ^∗∗^ *p*<0.05, ^∗∗∗^ *p*<0.01. Standard errors in parentheses.

Table A.4 shows the results of the small model (OLS, LASSO and Ridge methods) tested on the full dateset. The consolidated dataset is created by appending the main and the verification datasets. The results are virtually identical to the ones calculated on the main data (Table 6), with the sole exception of the violation of the minimum number of days for during which the call for tenders is published (*Open Tender Day V.*), for which the coefficient’s magnitude largely increases (consistently with the findings in the verification dataset) across all specifications. Nonetheless, the coefficient of *Open Tender Day V.* remains not statistically significant according to the OLS results. In terms of performance, the consolidated dataset has precision, recall and MSE values very similar to the ones of the main and of the verification data in all three models. In particular, the precision is slightly penalized in favor of a slightly higher recall.

**Table A.4:** Estimates for the small model—consolidated Data

	OLS	LASSO	Ridge
	Corruption risk	Corruption risk	Corruption risk
Design-Build	0.002	0.002	0.002
	[0.004]		
Urgency	−0.004	−0.004	−0.004
	[0.003]		
Negotiated	0.001	0.000	−0.001
	[0.004]		
Negotiated-No Tender	0.003	0.002	0.002
	[0.003]		
Price Only—w. ABA	−0.003	−0.006	−0.006
	[0.004]		
Scoring Rule (MEAT)	0.007^∗^	0.007	0.007
	[0.004]		
Open Tender Days	0.001	0.002	0.002
	[0.004]		
Open Tender Day V.	0.006	0.006	0.006
	[0.004]		
Observations	15,818	15,818	15,818
Adj R2	0.033		
MSE	0.355	0.127	0.127
False Positive	4399	4358	4322
False Negative	2726	2730	2737
Precision	0.223	0.225	0.225
Recall	0.317	0.316	0.314
Threshold	0.189	0.189	0.187

^∗^ *p*<0.1, ^∗∗^ *p*<0.05, ^∗∗∗^ *p*<0.01. All specifications include year and region fixed effects. Robust standard errors in parentheses for OLS estimates. Due to the limited number of observations in our sample, LASSO and Ridge regressions are evaluated through a standard k-fold cross-validation method (with *k* = 10), and not through the more common train-test split. MSE is equal to the root mean squared error for OLS, and to the minimal cross-validation mean squared error for LASSO and Ridge regressions. False Positive indicates the number of cases in which a non-corrupt firm is classified as corrupt by the model. False Negative indicates the number of cases in which a corrupt firm is classified as non-corrupt by the model. Threshold indicates the predicted value of the outcome variable for which a firm is classified a corrupt.

Below we also report performance graphs for the OLS, LASSO and Ridge models, to ensure full comparability the results across articles, and to compare the performance of the models when different thresholds are used. Figures A.1 to A.3 report Precision and Recall curves for the OLS, LASSO and Ridge models. Figures A.4 to A.6 report ROC curves for the same models.

Finally, in Fig. A.7, we report the result of running two random forest models, one for the dummies and one for the continous variables in our dataset. Continous variables tend to be more important than dummy variables in our specific case, however, when testing two separate models, one for the continous and one for the dummy variables, the relative importance of the variables presented in Fig. 1 is respected.

## Footnotes

1 However, we note that these findings are not univocal. It is also argued that in presence of red-tape, corruption may act as a grease in the wheel of bureaucracy, reducing costs of doing business and improving performance (Leff [43], Huntington [38], Dreher and Gassebner [28], Bologna and Ross [9]), and potentially leading to efficient allocation as the most productive firms may have the highest willingness to pay (Lui [45], Shleifer and Vishny [62], Weaver [69]).

2 Specifically, contracts for the building and maintenance of roads and highways in Italy, where corruption is considered a very relevant phenomenon (GRECO [34] and European Comission [29]).

3 See Fazekas, Toth and King [31] for a recent review of the use of red flags to detect corruption in public procurement. More generally, see Villamil, Kertész and Wachs [68] for a review of the more recent studies using computational methods to uncover corruption risk.

4 Clearly, scoring auctions are valuable tools to enhance a proper trade-off between cost and quality elements, making a procurement process cost-effective. However, as stated earlier, this study only looks at corruption predictors without attempting to evaluate such a complicated trade-off. See Coviello, Guglielmo and Spagnolo [20] and Decarolis et al. [22] for an empirical exploration of the trade-offs posed by scoring rule auctions and, more broadly, discretion in procurement.

5 Indeed, we also show that, by using the random forest model and the broader set of indicators, the ranking of red flags by importance differs from what is found with the alternative methods and specifications. For instance, while the scoring rule remains a significant predictor, features like the completeness of the documentation (measured in terms of the total number of document pages and words) become among the most important predictors, even though only marginally relevant under LASSO and RIDGE.

6 To some extent this applies to this study as well, especially if any public authority in charge of monitoring corruption were to adopt the list of red flags and prediction models presented in our analysis.

7 See Rizzica and Tonello [58] and Brodi, Occhilupo and Tonello [11] for a comprehensive review of the main problems.

8 Most times, such indexes derive from perceptions of the phenomena; other indexes originate from descriptions of broad aspects of governance and, as such, they mildly link with corruption itself. Examples of these indicators range from The Economist Intelligence Unit’s Business International Indicators to the World Bank Governance Indicators, the Transparency International Corruption Perceptions Index, the Global Corruption Barometer, and the European Commission Eurobarometer. See Fazekas, Toth and King [31] and Rizzica and Tonello [58] for an extensive review of these indicators and their use.

9 For example, see Mitchell and Campbell [49], who use federal corruption conviction rates in the U.S. as a measure of corruption, and Schulze, Sjahrir and Zakharov [60].

10 More recently, scholars have used data from random audits of governmental processes in Brazil to construct new measures of political corruption in local governments (Ferraz and Finan [32], Brollo et al. [12], Brollo and Troiano [13]) and corruption in public procurement contracts (Colonnelli and Prem [19]). While many of these studies have suggested more accurate and reliable methods of measuring corruption, their implementability remains limited as they are costly and difficult to replicate across countries.

11 In 2010, the World Bank issued a guide on the top ten most common red flags of fraud and corruption in procurement for bank-financed projects. At the European level, various policy projects have investigated the use of red flags to detect corruption and other illegal behaviors. An example is the European Commission Anticorruption Report (EU Commission, 2014, Report from the commission to the council and the European parliament-EU anticorruption report), aimed at starting a debate involving the European Commission, Member States, and other stakeholders to bolster corruption resolution and identify policies where a collective European effort can help. Within the EU Horizon 2020 framework, DIGIWHIST (http://digiwhist.eu/) brings together six European research institutes to empower society to combat public sector corruption through the systematic collection and analysis of information on public procurement in EU countries.

12 See Mullainathan and Spiess [52] and Athey [3].

13 The Italian public procurement sector of goods, services, and works mostly follows the European Union-wide regulations laid down in the EU Public Procurement Directives. In the period that we analyze these are Directives 2004/17/EC and 2004/18/EC, and the Italian law implementing these regulations is the Legislative Decree 12/4/2006, n. 163. See Decarolis, Giorgiantonio and Giovanniello [24] for an in-depth discussion of the national regulations and Decarolis and Giorgiantonio [23] for the local regulations. Italy has introduced relevant regulatory changes after 2016, when the Legislative Decree 18 April 2016, n. 50 implemented the current EU Procurement Directives (24/2014 and 25/2014). Among other things, the Legislative Decree of 2016 provided a reorganization of the functions of contracting authorities through greater centralization and the introduction of a unique qualification system to increase the professional specialization in the public contracts sector. However, this reorganization has not been implemented yet. We note that smaller municipalities (those that are not provincial capitals) had their ability to purchase goods and services over €40,000 and works over €150,000 reduced starting from the end of December 2015. Above these thresholds, these municipalities can merge their public procurement offices with those of either other municipalities or with their province procurement office or, for some purchases, relying on regional or national central purchasing bodies (see the Law Decree 24 April 2014, n. 66 and the Budget Law 2016). However, the Law 14 June 2019, n. 55 suspended these measures until the end of 2020.

14 E.g. The quality of the work or the time for completion. We should note that the 2014 European Directives on public procurement provided for the most economically advantageous tender criterion as the ordinary criterion for awarding public contracts.

15 Negotiated procedures, marked by significant discretionary powers for the administration, are those where the PAs consult their chosen economic operators and negotiate the conditions of the contract with one or more of them. Insofar as these procedures represent a derogation to the general ban on renegotiating offers, they are exceptional, being admissible (except for small amount contracts) only when specific conditions apply (chiefly those related to urgency or lack of appropriate offers or applicants).

16 An example of indicators is whether the solicitation procedure is negotiated (as opposed to a competitive auction), and one of its sub-indicators is whether the procedure involves or not the publication of a call for tenders.

17 E.g. investigations by the Sector Authority (Anticorruption Authority—ANAC) or judgments.

18 See Tribunal of Santa Maria Capua Vetere 26 March 2014, II Criminal Section, Picardi, President; Giovanniello, Extensor Judge. The Supreme Court confirmed this sentence in February 2019.

19 In 2000 the Sector Authority established a database on public works contracts. According to the Italian legislation, contracting authorities have to communicate a set of information related to each contract they award (e.g., value, awarding procedure and criterion, open tender days) to the Sector Authority. Our corruption indicators include data that have to be communicated by contracting authorities and data collected by a combination of human and machine learning analysis of the tender documentation (see paragraph 4). Hence, this latter is not directly available for the Sector Authority (currently, Anticorruption Authority).

20 A further potentially relevant dimension of classification could be on firms’ and the public authority’s awareness of monitoring of specific indicators. One could think that indicators that are known to be monitored by the Authority should have less predictive power. However, based on extensive conversations with those experts in the field of investigation that provided us with the data, we were told that, contrary to what outsiders might expect, there are no indicators specifically monitored and targeted by investigators.

21 In particular, in US Federal contracts for construction works worksite visits are typically required.

22 Contracts with a reserve price at or below 40,000 euros are not subject to systematic data collection by the Italian regulator, ANAC, from which we collected the data.

23 We collected this additional information by a combination of human and machine learning analysis of the tender documentation. The latter type of method was applied whenever feasible (for instance, to count the number of pages and words in the call for tenders). When we had to resort to human inspection, each document was scored by two persons, and any conflict resolved through expert legal advice. Although any entity supervising the sector takes steps analogous to the ones we undertook to collect the same data, the fact that these types of data are not readily available implies that market participants are aware of the non-systematic monitoring of these indicators.

24 Decarolis et al. [22] obtained this information for all firms ever involved in public works in the period between 2000 and 2017. They obtained it not only for firms winning the contracts but also for participants at the auctions and subcontractors, as well as for the public administrations handling the contracts. The collection of such data was possible thanks to a framework agreement between the agency for the internal intelligence and security under the Presidency of the Council of Ministers and Bocconi University.

25 Interestingly, this share of corruption risk winners is roughly constant independently of how we split the sample. Regardless of whether we use coarse measures of the location of the PA (i.e., North vs. South) or more sophisticated measures of the PA’s human capital from Baltrunaite et al. [4], among either its bureaucrats or its local ruling politicians, the proportion of corruption risk winners is always very similar and close to 15%.

26 Corruption cases are generally complex, and convictions relatively rare. This is particularly true in Italy, where the trial must go through three levels of judgment (*Primo grado*, *Appello*, and *Cassazione*) within a relatively short statute of limitation (between 6 and 12 years) considering the length of criminal proceedings. Only recently, the Law 9 January 2019, n. 3 provided for a lengthening of statutes of limitation, which entered into force from January 1st, 2020. We should note that the enforcement level can influence the novel measures of firm-level corruption risk that we use, but with a much more limited extent given that—*inter alia*—in these cases, statutes of limitations are not provided for.

27 The data on debarments is not publicly available. However, we obtained them through the related project of Decarolis et al. [22] previously described. The data contains each instance of *interdittiva*, *informativa* and *white list* denial.

28 As reported in Table 4, the variable *criminal* is imbalanced in our dataset, as only 15% of the observations have a positive *criminal* indicator. As reported in Provost [57] there are several risks that come with conducting a classification exercise on an imbalanced dataset. A possible solution, not followed in our study, would be to upsample or downsample our dataset. Downsampling would reduce the number of observations we have and this would be problematic for the verification data, especially if we consider that missing values are present across features. Furthermore, since a goal of this paper is to propose a simple and standardized ML approach, throughout the analysis we tried to limit all of those steps that are less standardized, as it is the case for the problem of addressing unbalancedness. Nevertheless, we acknowledge that evaluating the effects of the upsample or downsample strategies proposed in the literature might be a valuable extension of our work.

29 “Prediction policy problems”, see Kleinberg et al. [42].

30 Indeed, we see model selection as useful by itself in the spirit of designing and using statistical screens, Abrantes-Metz and Bajari [1].

31 This logic does not apply to all indicators, some of which might have more profound effects on the behavior and incentives of the agents involved. We return to this issue after having presented the results.

32 Following Athey [3], ML methods can be described as algorithms that might estimate many alternative models and then select among them to maximize a criterion. There are a plethora of ML methods for supervised learning. The most common ones include regularized regression (LASSO, Ridge and elastic net), regression trees, ensemble methods (random forest), neural networks, matrix factorization, and support vector machines. For an excellent overview of many of these methods and their applications to economics, see Varian [67], Mullainathan and Spiess [52] and Athey [3].

33 See Ng [54], Chernozhukov et al. [17] and Primiceri, Giannone and Lenza [56]. Sparse-models select a relatively small set of regressors that maximize predictive power. The most popular method among sparse modeling techniques is the LASSO (Tibshirani [64], Tibshirani, Wainwright and Hastie [65], Belloni, Chernozhukov and Wang [8]). On the other side, dense models aim at keeping all possible regressors in the model but solve the over-fitting problems by reducing the size of the parameter estimates whenever the sample information reaches a lower bound. These methods are usually known as “shrinkage” or “regularizing” methods. Ridge regression is the most common example.

34 Random Forest is an ensemble tree-based learning algorithm, meaning that the final prediction model is an average of the predictions obtained growing many individual trees. The algorithm uses bootstrap aggregating, also known as bagging, to limit over-fitting and improve out-of-sample accuracy. Bagging implies fitting each tree on a bootstrap sub-sample, rather than on the original full training sample. The method consists of the following steps. First, a given number of random sub-samples are drawn from the training sample. Second, a random subset of variables among the entire set of predictors is selected. This subset of variables is used to determine each subsequent split in a tree. Each internal node is split until a certain predictor optimizes the splitting criterion. Then, several decision trees are grown, one for each randomly-drawn sample. Each decision tree is built up to its maximum size (no pruning occurs). This leads to a very dense forest. Finally, the trees are combined, averaged.

35 We report in the text the results using Stata15 routines of Townsend [66] for LASSO and Ridge and of Schonlau [59] for the random forest. Both Stata and Python codes will be made available on the authors’ web site.

36 For instance, as we mentioned before (Sect. 3), the 2014 European Directives on public procurement provide for the MEAT criterion as the ordinary criterion for awarding public contracts.

37 We note that, in the period of our analysis (2009–2015), the threshold within which negotiated procedures can be used for awarding public works contracts even if specific urgency conditions do not apply, was gradually raised from €200,000 to €1,000,000. These regulatory changes may have reduced the necessity of invoking an urgency to use faster procedures and greater flexibility.

38 It is essential to emphasize that the ML literature does not frame itself as aimed at solving estimation problems. Hence, estimating $\Pr (Y = k|X = x)$ is not the primary goal. Instead, the goal is to achieve goodness of fit in an independent test set by minimizing deviations between actual outcomes and predicted outcomes. In applied econometrics, we often wish to understand an object like $\Pr (Y = k|X = x)$ to perform exercises like evaluating the impact of changing one covariate while holding others constant. This is not an explicit aim of ML modeling. LASSO and Ridge regression are the only exception.

39 This is mainly due to *a*) incomplete communications by the contracting authorities in the tender documents or *b*) problems in the match with the complementary ANAC data on some outcomes and controls. While it would be impossible to run estimation on a subsample of tenders for which we have no missing across all variables (as we would end up with fewer observations than variables), we proceed by filling in the missing values in the following way. For each indicator, we replace the missing value with the median value across the sample. We then create a dummy variable tracking the filling procedure for that particular variable. We thus include 20 extra dummy variables in our regressions.

40 This might be useful in order to select only a subset of variables to use in a standard regression model, or—in general—to have a sense of which are the most critical drivers of the observed outcomes. In terms of the train/test split, we use a recurring binary hierarchical splitting process.

41 In the Appendix, we further explore this heterogeneity across different data types by reporting in Fig. A.7 the outcomes of the random forest when we run the model separately for the cases in which all of the included red flags are either continuous or Boolean.

42 Athey [3] foresees a steady increase in the connection between ML and economics methods. Such a process involves using the ML methods to conduct an initial model selection phase that is currently done in an informal and undocumented way, but that might refute the validity of the estimates via problems of specification search and multiple testing. In this sense, our work can be seen as indeed part of a broader research project that continues in other works where we analyze through conventional economic methods the effects of more discretionary awarding procedures and criteria on political connections (Baltrunaite et al. [4]) and corruption (Decarolis et al. [22]).

43 Following [44] these conditions (SANS conditions) that are useful in identifying whether the results obtained from a narrow, specific reform (or experiment) are sufficiently likely to hold in broader contexts are: (i) representativeness of the sample with respect to the full population and representativeness of the sample with respect to the relevant variables for the study; (ii) attrition rates and reasons for attrition and non compliance; (iii) naturalness of the setting, choices, tasks and time frame observed; and (iv) scalability of the results, cost-benefit of the policy proposed at scale and conditions that would affect the outcomes.

44 While not aware of cases for corruption detection via red flags, we note there are successful stories regarding collusion. For instance, Imhof, Rutz and Karagök [40] discuss a case of a cartel of firms bidding on roadwork procurement auctions in Switzerland that was initially detected through statistical screens and, later, convicted by the court once hard evidence on this cartel emerged.

45 Furthermore, this is an interesting instance of the practice anticipating the theory as very recent by Chassang et al. [15] has offered theoretical foundations and further empirical verification precisely for the main red flags that triggered the Swiss case.

## Abbreviations

ANAC: Italian Anticorruption Authority
EU: European Union
GDP: gross domestic product
MEAT: most economically advantageous tender
ML: machine learning
MSE: mean squared error
OP: operating practices
